# Neuroprotective Effects of Anodal tDCS on Oxidative Stress and Neuroinflammation in Temporal Lobe Epilepsy

**DOI:** 10.3390/biomedicines14010023

**Published:** 2025-12-22

**Authors:** Ali Osman Arslan, Sevdenur Akcay, Guven Akcay, Dana Zaqzouq, Aydın Him

**Affiliations:** 1 Department of Medical Biology, Faculty of Medicine, Bolu Abant İzzet Baysal University, Bolu 14030, Turkey; aliosmanarslan@ibu.edu.tr; 2 Department of Physiology, Faculty of Medicine, Bolu Abant İzzet Baysal University, Bolu 14030, Turkey; sevdenuruzunn@gmail.com (S.A.); danov_y@yahoo.com (D.Z.); aydinhim@hotmail.com (A.H.); 3 Department of Biophysics, Faculty of Medicine, Bolu Abant İzzet Baysal University, Bolu 14030, Turkey

**Keywords:** epilepsy, learning and memory, neuroinflammation, pentylenetetrazole (PTZ), transcranial direct current stimulation (tDCS)

## Abstract

**Background:** Epilepsy affects over 50 million people worldwide, and about 30% remain drug-resistant—underscoring the urgent need for new therapies. This study evaluated the neuroprotective effects of anodal transcranial direct current stimulation (tDCS) in PTZ-induced epilepsy at acute and chronic stages in rats. **Methods:** Sixty male Wistar Albino rats (12 per group) were randomly assigned to five groups: control, acute epilepsy, acute epilepsy+ tDCS, chronic epilepsy, and chronic epilepsy+ tDCS. Behavioral tests—including the open-field, novel-object recognition, and Y-maze—assessed locomotion, recognition, and spatial memory. Hippocampal tissues were analyzed for oxidative stress markers (SOD, MDA), inflammatory cytokines (IL-1β, TNF-α), histopathology, and mechanistic markers of astrocytic and nitric oxide-mediated neuronal damage (GFAP and nNOS immunohistochemistry). **Results:** PTZ-induced epilepsy resulted in cognitive deficits, increased oxidative stress, neuroinflammation, neuronal degeneration, and astrocytic activation. Specifically, SOD decreased, while MDA, IL-1β, and TNF-α increased; GFAP and nNOS upregulation indicated activation of astrocytes and nitric oxide-mediated neuronal damage. tDCS mitigated these effects by enhancing SOD, reducing MDA, IL-1β, and TNF-α, and modulating the NO/GFAP axis, which corresponded to decreased neuronal degeneration and vascular hyperemia. Behaviorally, tDCS improved recognition memory and partially rescued spatial memory deficits. **Conclusions**: Anodal tDCS exerts neuroprotective effects in acute and chronic epilepsy by modulating oxidative stress, neuroinflammation, and the astrocytic/nitric oxide pathways, supporting its potential as a non-invasive adjunct therapy for cognitive and cellular protection. Future studies should investigate its effects on hippocampal glutamatergic and GABAergic pathways, as well as calcium homeostasis.

## 1. Introduction

Epilepsy ranks among the most common and impactful neurological conditions globally, influencing over 50 million people and creating significant physical, mental, social, and economic challenges [1,2]. Epilepsy is characterised by recurrent and un-provoked seizures resulting from a complex interplay of neuronal hyper-excitability, disturbed synaptic inhibition/excitation balance, glial dysfunction (notably astrocytic dysregulation), ionic channel abnormalities, neuroinflammation and oxidative stress [3]. Patients with drug-resistant epilepsy (approximately 30% of cases) remain refractory to antiseizure medications, underscoring the need for novel non-invasive neuromodulatory therapies. Recent meta-analyses (2023–2024) indicate that transcranial Direct Current Stimulation (tDCS) significantly reduces seizure frequency in refractory epilepsy and is well tolerated [4,5]. These findings support the potential of tDCS to intervene not only at the behavioural level but also at mechanistic targets such as astrocyte activation (e.g., GFAP), nitric-oxide signalling (nNOS) and oxidative/inflammatory cascades, thereby bridging electrophysiological hyper-excitation and supportive glial/oxidative pathomechanisms. Although the pathophysiology of epilepsy remains incompletely understood, disturbances in the interplay of excitatory glutamate (GLU) and inhibitory gamma-aminobutyric acid (GABA) have been implicated, with elevated GLU activity identified as a central mechanism in epileptogenesis [6].

Experimental epilepsy models, such as pentylenetetrazole (PTZ) kindling, induce neurochemical and structural alterations, including hippocampal atrophy, neuronal loss, and astrocytosis [7]. PTZ-induced seizures elevate nitric oxide (NO) production via neuronal nitric oxide synthase (nNOS), resulting in oxidative stress and neuroinflammation [8,9]. Higher concentrations of GFAP, a recognized marker of astrocyte activation, have been detected in both epilepsy and several neurodegenerative disorders [10,11]. Furthermore, TNF-α, IL-1β, and IL-6, as pro-inflammatory signaling molecules, significantly contribute to the development of epilepsy by inducing neuronal damage and astrocyte activation [12].

Oxidative stress and mitochondrial dysfunction play a central role in the pathophysiology of epilepsy, with increased production of free radicals and lipid peroxidation further exacerbating neuronal damage. Post-seizure, markers such as malondialdehyde (MDA) show a significant increase, whereas antioxidant defenses, including superoxide dismutase (SOD) and glutathione (GSH), are markedly reduced [12,13].

tDCS, a noninvasive neuromodulation approach, is increasingly recognized as a promising intervention for a range of neurological diseases, including seizure disorders, stroke, and depressive disorders [14,15,16,17]. By modulating cortical excitability via Na^+^-Ca^2+^ channels and NMDA receptor activity, tDCS induces neuroplastic changes and affects both GABAergic and glutamatergic synapses [18,19]. Notably, tDCS represents a safe and cost-effective therapeutic option, with minimal side effects such as transient headaches or mild skin irritation [20]. Anodal tDCS increases cortical excitability, whereas cathodal stimulation exerts inhibitory effects, with these outcomes often persisting for extended periods post-treatment [18,21].

In pharmacoresistant epilepsy, affecting roughly 20–30% of patients who show little or no response to antiepileptic medications, neuromodulation therapies such as tDCS provide a complementary treatment option [22,23]. tDCS has shown potential in mitigating epilepsy-related cognitive impairments, particularly in learning and memory, which represent some of the most critical concerns for patients with epilepsy [24,25].

This study assessed how tDCS influences therapeutic outcomes in acute and chronic epilepsy models. Behavioral and molecular parameters were assessed to elucidate the neuroprotective mechanisms of tDCS, with particular focus on its role in mitigating oxidative stress, attenuating neuroinflammation, and enhancing cognitive function.

## 2. Materials and Methods

The experiments were performed at the Experimental Animals Unit of Akdeniz University using rats supplied by the university’s Experimental Animals Application and Research Center. The experiments were performed in accordance with the approved guidelines and regulations. All experiments were performed in accordance with the approved guidelines and regulations. The reporting of this animal research follows the ARRIVE guidelines (https://arriveguidelines.org, accessed on 2 December 2025).

### 2.1. Experimental Protocol

**Figure 1:**
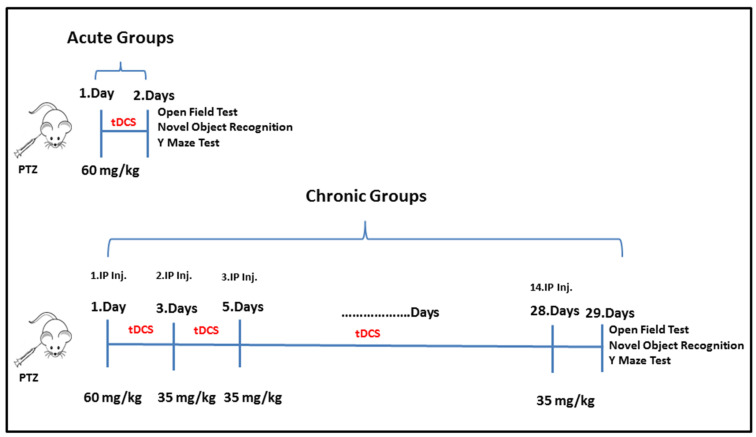
Experimental protocol.

Sixty male Wistar Albino rats, three months old with a body weight of 250–300 g, were employed for the experiments. Using randomization and blinding procedures, a total of 60 rats were placed into five experimental groups, with 12 animals per group: control and epilepsy models for both acute and chronic epilepsy, as well as corresponding epilepsy groups receiving tDCS treatment. Temporal lobe epilepsy (TLE) was induced using PTZ. The acute group received 30-min 1 mA anodal tDCS stimulation for the first two days following PTZ administration, while the chronic group received tDCS treatment on alternate days (2, 4, 6, 8, 10, …, 26 and 28) without PTZ administration. Behavioral evaluations, including the open field (OF), novel object recognition (NOR), and Y-maze tasks, were conducted to assess locomotor function, recognition abilities, and spatial learning, respectively. After the behavioural experiments, animals were euthanized by cervical dislocation on day 3 after behavioral experiments, the brains were removed, and hippocampal tissue was collected for biochemical and histopathological analyses. Immunohistochemical analyses were performed for neuronal nitric oxide synthase (nNOS) and glial fibrillary acidic protein (GFAP), whereas oxidative stress and inflammatory parameters—including SOD, MDA, IL-1β, and TNF-α—were quantified via ELISA. The detailed experimental timeline is shown in Figure 1.

### 2.2. Experimental Model of PTZ-Induced Epilepsy

To induce acute epilepsy, rats received a single intraperitoneal injection of PTZ at a dose of 60 mg/kg. For the chronic TLE model, an initial PTZ dose of 60 mg/kg was followed by subsequent injections of 35 mg/kg administered every other day until animals consistently exhibited Racine stage 5 seizures [26]. This dosing regimen allowed for the reliable establishment of both acute and chronic epileptic phenotypes, enabling subsequent behavioral, molecular, and histopathological assessments.

### 2.3. tDCS Application

Anodal tDCS (1 mA, 30 min) was delivered via a superficial disc electrode using a DCS stimulator designed for animals (model 2100), which allows precise current control (±1000 μA) and temporal accuracy (1 min). Stimulation was applied 4 h post-PTZ injection for the acute groups and on non-injection days for chronic groups. The tDCS protocol is identical to the protocol used in our previous study, which demonstrated its effects on the hippocampal region in an experimental stroke model [17]. To investigate the effects of tDCS, the anodal electrode was positioned between the ears, from the neck of the rat (parietal cortex), while the cathodal electrode was positioned at the midpoint of the lateral angle of the eyes (supraorbital area) [17].

### 2.4. Behavioral Experiments and Biochemical Analysis in Rodent Models

#### Assessment of Locomotor Activity: Open Field Test (OF)

The animals’ locomotor behavior was assessed with the OF test in an arena measuring 80 × 80 cm, featuring walls 40 cm in height and 16 squares (20 cm × 20 cm each) delineated on a black surface. Rats were introduced into the middle four squares and given 5 min to explore the arena. Distance traveled (cm) and the number of squares traversed were monitored using the video camera. Between each trial, the apparatus was sanitized to eliminate residual olfactory or visual cues [27,28].

### 2.5. Evaluation of Recognition Memory Using the Novel Object Recognition (NOR) Test

The Novel Object Recognition (NOR) paradigm, commonly employed to evaluate attention and short-term memory, was conducted in three phases: habituation, training, and retention. For habituation, rats were allowed to explore a 40 × 40 cm arena without objects for 5 min. During the training phase, a pair of identical objects was positioned in the arena, and the rats were allowed to explore for 5 min. During the retention phase, one familiar object was substituted with a new object, and the time spent exploring it was measured over 5 min. The duration of exploration directed toward the novel object (in seconds) was used as a measure of recognition memory performance [29].

### 2.6. Assessment of Spatial Memory: Y-Maze Test

Spatial memory was evaluated using a Y-maze paradigm consisting of two sessions in a Y-shaped apparatus with three identical arms (50 × 20 × 10 cm) positioned 120° apart. During the initial session, the “novel arm” was kept closed, and rats were permitted to explore the remaining two arms for 15 min. After a one-hour interval in their home cages, the second session permitted free exploration of all three arms for 5 min. Entries into the novel arm and the duration of time spent there were measured using the video camera. Successful spatial memory was indicated by preference for the novel arm [30].

### 2.7. Tissue Collection and Storage

Animals were euthanized by cervical dislocation after behavioral experiments, and tissue samples were obtained. Collected tissues were kept at −80 °C for subsequent biochemical and histological analyses.

### 2.8. Biochemical Analysis: MDA, SOD, TNFα, and IL 1β Assays

Hippocampal samples were homogenized and centrifuged (4500 rpm, 4 °C, 10 min), after which the supernatants were examined to determine malondialdehyde (MDA), superoxide dismutase (SOD), tumor necrosis factor-alpha (TNF-α), and interleukin-1 beta (IL-1β) levels using commercially available sandwich ELISA kits (R&D Systems, Minneapolis, MN, USA). The biochemical analyses of MDA, SOD, TNF-α, and IL-1β were normalized to total protein content per mg of tissue, which was determined using the Bradford assay with bovine serum albumin as the standard.

### 2.9. Histopathological Analysis

Brain tissues were fixed in 10% formalin for 48 h, then dehydrated through ascending alcohol concentrations and cleared with xylene. Following paraffin embedding, the samples were sliced into 4 μm sections and stained with hematoxylin and eosin (HE) for histological analysis. Slides were analyzed under a light microscope (Olympus BX51, Japan), and histopathological findings were scored as none (−), mild (+), moderate (++), or severe (+++).

### 2.10. Immunohistochemical Detection: Glial Fibrillary Acidic Protein (GFAP)

Immunohistochemical analysis of GFAP was performed on tissue sections embedded in paraffin and mounted on poly-L-lysine–coated slides. After deparaffinization, antigen retrieval in citrate buffer (pH 6.1) was performed using microwave heating. Sections were incubated with primary anti-GFAP antibodies (Cat. No: ab68428; dilution 1:100, Abcam, UK) followed by biotinylated secondary antibodies and streptavidin-peroxidase. Chromogen staining was conducted with DAB, and counterstaining was performed with Mayer’s hematoxylin. Samples were graded based on immunopositivity as none (−), mild (+), moderate (++), or severe (+++).

### 2.11. Immunofluorescence Analysis: Neuronal Nitric Oxide Synthase (nNOS)

For nNOS immunofluorescence, tissue sections were placed on poly-L-lysine–coated slides, deparaffinized, and processed for antigen retrieval. Primary anti-nNOS antibodies (Cat. No: ab76067; dilution 1:100, Abcam, UK) were applied, followed by FITC-conjugated secondary antibodies (Cat. No: ab6785; dilution 1:1000, Abcam, UK). Fluorescently labeled sections were examined using a Zeiss AXIO fluorescence microscope (Germany) and evaluated for immunopositivity as none (−), mild (+), moderate (++), or severe (+++).

### 2.12. Statistical Analysis

Histopathological findings were evaluated using the nonparametric Kruskal–Wallis test, and pairwise comparisons were performed with the Mann–Whitney U test. Biochemical and behavioral data were analyzed through one-way ANOVA followed by Tukey’s multiple comparison test. A *p*-value below 0.05 was considered statistically significant. All analyses were conducted using SPSS software, version 13.0 (SPSS Inc., Chicago, IL, USA).

## 3. Results

### 3.1. Impact of tDCS on Locomotion, Spatial Learning, and Memory in Acute and Chronic Groups

**Figure 2:**
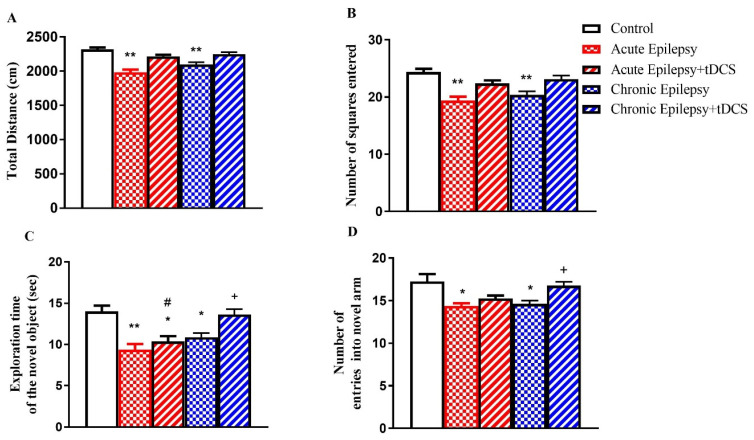
Behavioral outcomes in the acute and chronic groups. (**A**) Total distance covered (cm) in the open field (OF) test, (**B**) number of squares traversed in the OF, (**C**) duration of novel object exploration (sec) in the novel object recognition (NOR) test, and (**D**) number of entries into the novel arm in the Y-maze task. Data represent mean ± SEM (n = 12 per group). Statistical significance: * *p* < 0.05, ** *p* < 0.01 vs. control; # *p* < 0.05 vs. acute epilepsy group; + *p* < 0.05 vs. chronic epilepsy group (one-way ANOVA with Tukey’s multiple comparison test).

Locomotor behavior during the open field test, including total distance (cm) and number of squares entered, is depicted in Figure 2A,B. Although the acute epilepsy, acute epilepsy + tDCS, chronic epilepsy, and chronic epilepsy + tDCS groups showed reductions relative to controls, a statistically significant decline was detected only in the acute epilepsy and chronic epilepsy groups (*p* < 0.05). In the NOR paradigm, the time spent exploring the novel object is shown in Figure 2C. Statistically significant reductions were detected in the acute epilepsy (*p* < 0.001), acute epilepsy + tDCS (*p* < 0.05), chronic epilepsy (*p* < 0.05), and chronic epilepsy + tDCS (*p* < 0.05) groups compared with controls. However, the acute epilepsy + tDCS group exhibited a statistically significant enhancement (*p* < 0.05) relative to the acute epilepsy group, and the chronic epilepsy + tDCS group displayed a significant improvement (*p* < 0.05) compared with the chronic epilepsy group. Spatial memory, assessed via the Y-maze test, is shown in Figure 2D. A significant decrease in entries to the novel arm was observed in the acute epilepsy and chronic epilepsy groups relative to the control group. The acute epilepsy + tDCS group showed a nonsignificant reduction relative to controls and a minor, nonsignificant improvement versus the acute epilepsy group. Similarly, the chronic epilepsy + tDCS group exhibited a nonsignificant reduction relative to controls and a minor, nonsignificant improvement versus the chronic epilepsy group.

### 3.2. Levels of SOD, MDA, IL-1β, and TNF-α in Acute and Chronic Experimental Groups

**Figure 3:**
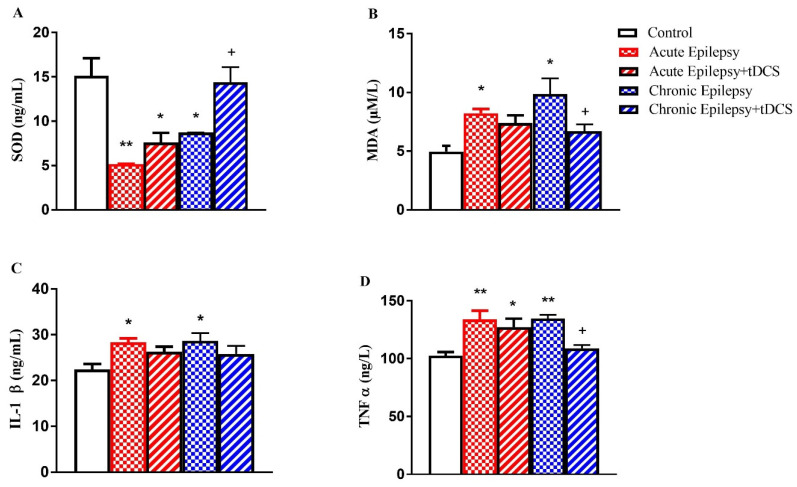
Oxidative stress and neuroinflammatory markers in the acute and chronic groups. (**A**) Superoxide dismutase (SOD), (**B**) malondialdehyde (MDA), (**C**) interleukin-1β (IL-1β), and (**D**) tumor necrosis factor-α (TNF-α) concentrations. Data are presented as mean ± SEM (n = 8 per group). Statistical significance was evaluated using one-way ANOVA followed by Tukey’s post hoc analysis (* *p* < 0.05, ** *p* < 0.01 vs. control; *p* < 0.05 vs. acute epilepsy group; + *p* < 0.05 vs. chronic epilepsy group).

Figure 3 illustrates the levels of biochemical indicators related to oxidative stress and neuroinflammatory responses in both acute and chronic experimental groups. SOD concentrations were significantly lower in the acute epilepsy, acute epilepsy + tDCS, and chronic epilepsy groups relative to the control group (*p* < 0.01, *p* < 0.05, and *p* < 0.05, respectively; Figure 3A). In contrast, concentrations of MDA, IL-1β, and TNF-α were significantly higher in both the acute and chronic epilepsy groups (*p* < 0.05, *p* < 0.05, and *p* < 0.01, respectively). In the acute and chronic epilepsy + tDCS groups, SOD levels were increased, while the concentrations of MDA, IL-1β, and TNF-α were reduced compared to the corresponding epilepsy groups, indicating a modulatory effect of tDCS on oxidative stress and neuroinflammatory responses.

### 3.3. Acute and Chronic Groups Histopathological

**Figure 4:**
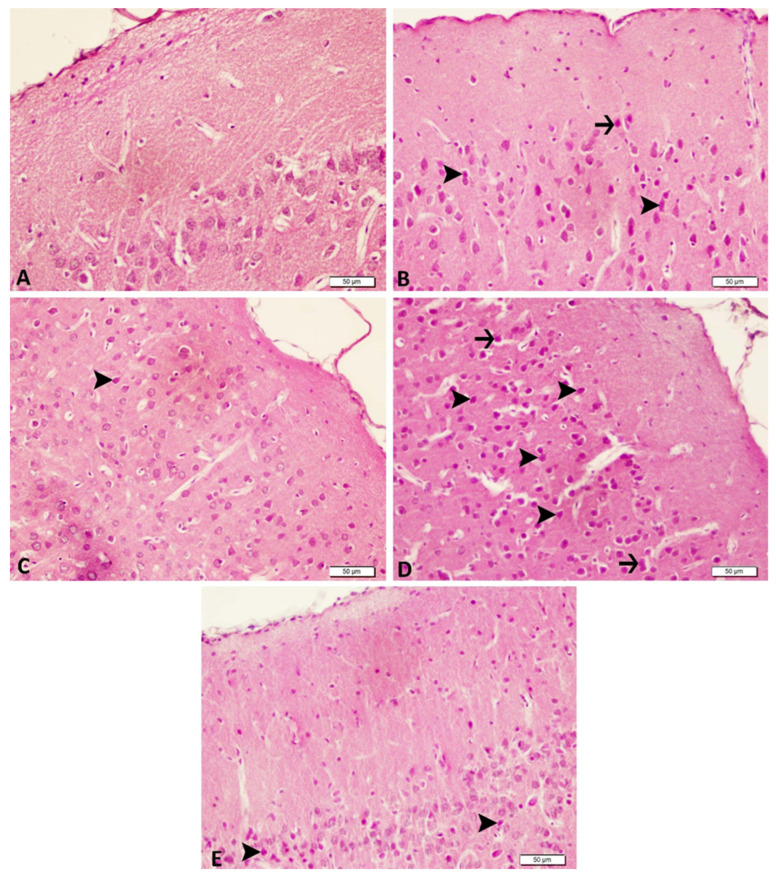
Histopathological Results. Hippocampal region: In the Control group, hippocampal histology appears normal (**A**). The Acute Epilepsy group exhibits moderate neuronal degeneration (arrowheads) accompanied by necrotic changes (arrows) (**B**), whereas tDCS treatment in this group reduces degeneration to a mild level (arrowheads) (**C**). In Chronic Epilepsy, severe neuronal degeneration (arrowheads) and necrosis (arrows) are observed (**D**), while Chronic Epilepsy + tDCS results in moderate neuronal degeneration (arrowheads) (**E**). Each group includes n = 4 animals; H&E staining; scale bar = 50 µm.

Histopathologic and histologic evaluations revealed normal brain tissue architecture in the control group (Figure 4A). Examination of the acute epilepsy group revealed notable neuronal degeneration, moderate necrotic changes, and prominent hyperemia within meningeal and parenchymal vessels (Figure 4B). In the chronic epilepsy group, moderate neuronal degeneration, mild necrotic changes, and moderate vascular hyperemia were observed (Figure 4B). In the tDCS-treated groups, moderate neuronal degeneration and vascular hyperemia were observed in the acute epilepsy group receiving tDCS (Figure 4C, while milder neuronal degeneration and vascular hyperemia were observed in the chronic epilepsy + tDCS group (Figure 4D,E). These findings reflect the protective effect of tDCS in reducing neuronal damage. Detailed findings are presented in Table 1.

### 3.4. Acute and Chronic Groups Immunohistochemical Findings (GFAP)

**Figure 5:**
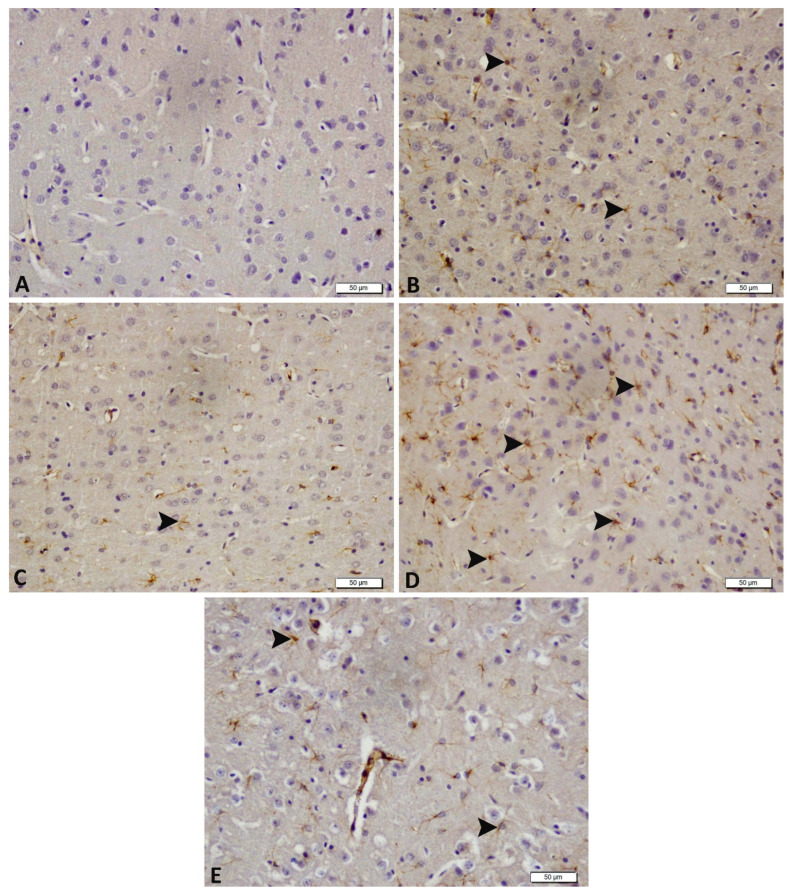
Immunohistochemical Results (GFAP). Hippocampal region: Control group, astrocytes showed no GFAP expression (**A**); Epilepsy group, astrocytes displayed moderate GFAP immunoreactivity (arrows) (**B**); Epilepsy + tDCS group, astrocytes exhibited mild GFAP expression (arrows) (**C**); Chronic Epilepsy group, astrocytes showed strong GFAP expression (arrows) (**D**); Chronic Epilepsy + tDCS group, astrocytes demonstrated moderate GFAP expression (arrows) (**E**). n = 4 per group; scale bar = 50 µm.

Immunohistochemical analysis revealed negative GFAP expression in the control group (Figure 5A). Moderate GFAP expression was noted in both acute and chronic epilepsy groups (Figure 5B,D), whereas tDCS-treated groups (acute and chronic epilepsy + tDCS) exhibited mild GFAP expression (Figure 5C,E). These findings suggest that tDCS may reduce astrocytic activation linked to acute and chronic epilepsy and are summarized in detail in Table 1.

### 3.5. Acute and Chronic Groups Immunofluorescence Findings (nNOS)

**Figure 6:**
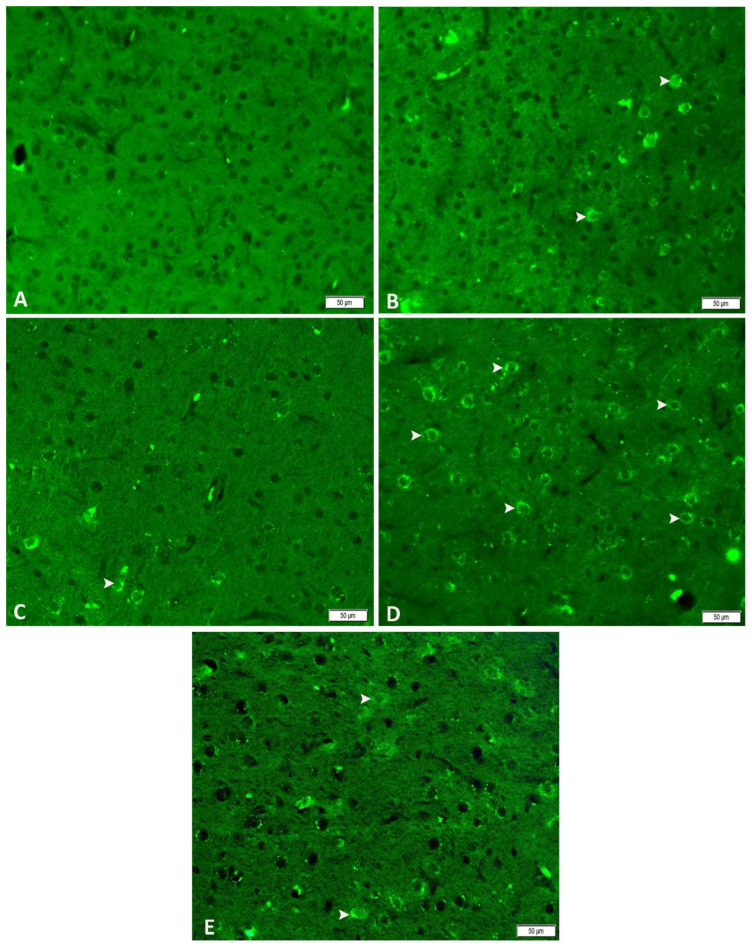
Immunofluorescence staining with DAPI (nNOS Hippocampal region: In the control group, nNOS expression was absent (**A**); in the acute epilepsy group, moderate nNOS expression was detected (arrows) (**B**); the acute epilepsy + tDCS group showed mild nNOS expression (arrows) (**C**); the chronic epilepsy group exhibited severe nNOS expression (arrows) (**D**); and the chronic epilepsy + tDCS group demonstrated moderate nNOS expression (arrows) (**E**). n = 4 per group, assessed by immunofluorescence (IF).

Immunofluorescence analysis revealed negative nNOS expression in the control group (Figure 6A). Severe nNOS expression appeared in the acute epilepsy group (Figure 6B), whereas a moderate reduction was noted in the acute epilepsy group treated with tDCS (Figure 6C). In the chronic epilepsy group, moderate levels of nNOS expression were recorded (Figure 6D), but, in the epilepsy + tDCS group, it decreased to mild levels (Figure 6E) (Table 1). These findings suggest that tDCS may attenuate neuronal nitric oxide synthase-induced neuroinflammatory damage by modulating nNOS activity.

The results collectively demonstrate that tDCS can attenuate some of the behavioral, biochemical, histopathological and molecular impairments associated with acute and chronic epilepsy. These findings underscore the potential of tDCS as an adjunctive therapeutic strategy in acute and chronic epileptic conditions.

## 4. Discussion

Epilepsy, a neurological disorder impacting more than 50 million individuals globally and accounting for approximately 2.4 million new cases each year, poses a substantial global health burden. Importantly, nearly 30% of individuals with epilepsy do not respond to antiepileptic medications, emphasizing the necessity for alternative treatment approaches. Non-invasive neuromodulation techniques, including tDCS, have shown considerable potential as therapeutic interventions. The pathogenesis of epilepsy is closely linked to disturbances in GABAergic and glutamatergic neurotransmission, leading to intracellular calcium buildup, triggering apoptotic and inflammatory signaling, and causing subsequent neuronal injury. Seizure-induced oxidative stress predominantly affects hippocampal subfields CA1, CA3, and dentate gyrus, which are crucial for memory and spatial cognition. Elevated MDA and reduced SOD in these regions drive inflammatory cytokine cascades (IL-1β, TNF-α) and astrocytic activation, contributing to neuronal dysfunction. Anodal tDCS mitigates these oxidative and inflammatory alterations, supporting its neuroprotective effect in TLE-associated cognitive impairment. The present study evaluated the impact of transcranial direct current stimulation (tDCS) on oxidative stress, inflammatory responses, and behavioral as well as molecular changes, with a particular focus on hippocampal markers such as nNOS, GFAP, SOD, MDA, IL-1β, and TNF-α [31,32]. PTZ-induced epilepsy increased MDA and decreased SOD, which was accompanied by upregulation of IL-1β and TNF-α, indicating that oxidative stress amplifies inflammatory cytokine cascades. Anodal tDCS restored redox balance and attenuated cytokine expression, revealing mechanistic crosstalk between oxidative and inflammatory pathways underlying neuroprotection.

The experimental TLE model was developed based on the methodology of Liao et al. (2016) [26], with acute TLE induced by a single intraperitoneal injection of 60 mg/kg PTZ, whereas chronic TLE consisted of an initial 60 mg/kg PTZ injection on day one, followed by 35 mg/kg PTZ on subsequent alternate days. PTZ is administered every other day until seizure ignition. TLE, accounting for 50% of epilepsy cases, predominantly affects the hippocampus, a critical structure for episodic learning and memory [33]. Previous studies have implicated neurodegeneration, synaptic plasticity alterations, and gliosis in the cognitive impairments associated with TLE [34]. Consistent with these results, this study revealed notable deficits in cognitive functions, including learning and memory, in both acute and chronic epilepsy models induced by PTZ. Notably, 13 days of anodal tDCS stimulation (1 mA for 30 min daily) ameliorated these deficits, exhibiting a neuroprotective effect.

Oxidative stress, a hallmark of epilepsy pathophysiology, involves elevated lipid peroxidation and free radical production. MDA, a marker of lipid peroxidation, increases following PTZ-induced seizures, while intracellular antioxidants such as SOD decrease [12,13]. In this study, PTZ-induced epilepsy significantly elevated MDA levels and reduced SOD activity in both acute and chronic models. Remarkably, tDCS treatment reversed these effects, with significant increases in SOD activity and reductions in MDA levels, particularly in chronic epilepsy + tDCS groups. Furthermore, inflammatory pathways, characterized by elevated TNF-α and IL-1β levels, were modulated by tDCS, demonstrating reductions in these pro-inflammatory markers in both acute and chronic epilepsy models. This aligns with findings that TNF-α mediates synaptic reorganization, glutamate dysregulation, and GABA receptor endocytosis, contributing to epileptogenesis [12,35].

Astrocytic activation, indicated by increased GFAP expression, is a hallmark of neuronal injury in epilepsy [10,11]. In this study, PTZ-induced epilepsy significantly upregulated GFAP expression in acute and chronic models. tDCS stimulation attenuated GFAP expression, highlighting its potential to mitigate astrocytic activation. Similarly, nNOS, a key mediator in nitric oxide production and epilepsy-related neuronal damage, was significantly elevated in PTZ-induced epilepsy. tDCS treatment reduced nNOS expression in the acute as well as chronic epilepsy models, further supporting its neuroprotective role [9,36].

The excitability of cortical neurons is elevated by anodal tDCS, which depolarizes neuronal membranes through the engagement of Na^+^- and Ca^2+^-dependent ion channels [18]. Accordingly, glutamate and gamma-aminobutyric acid (GABA) were chosen for investigation in this study because of their critical roles in mediating the effects of tDCS treatment. Furthermore, tDCS was selected due to its ability to influence the activity of ionotropic glutamate receptors, including NMDA and AMPA subtypes. Previous research from our group has demonstrated that tDCS exerts a modulatory influence on calcium- and glutamate-mediated excitotoxicity [15,16,37]. Furthermore, tDCS has exhibited protective and treatment-related effects in epilepsy by modulating the expression of TAS, TOS, Ca^2+^, glutamate, GABA, AMPAR1, and NMDAR1 [14]. This is another reason why anodal tDCS was selected for this study. The present research assessed the impact of tDCS on hippocampal oxidative stress and neuroinflammatory markers in rat models of acute and chronic TLE.

Previous studies have demonstrated the efficacy of electrical stimulation in modulating GABA and glutamate release and reducing seizure severity. For instance, Santana-Gómez et al. (2015) demonstrated that focal transcranial electrical stimulation attenuated hippocampal glutamate release and increased GABA release in a pilocarpine-induced epilepsy model [38]. Similarly, Regner et al. (2020) reported that cathodal tDCS combined with diazepam reduced neuroinflammation without significantly affecting seizure activity in PTZ-induced epilepsy [39]. In accordance with previous results, our research showed that anodal tDCS not only improved cognitive function but also reduced markers of oxidative stress, inflammation, and astrocytic activation.

In conclusion, tDCS treatment demonstrated notable neuroprotective and therapeutic effects in both early and long-term epilepsy models by reestablishing redox and inflammatory homeostasis and attenuating neuroinflammatory responses. This study highlights the potential of tDCS as a non-invasive intervention for mitigating epilepsy-related cognitive impairments and cellular damage. Future research will focus on elucidating the effects of tDCS on hippocampal glutamatergic and GABAergic pathways, particularly their roles in calcium homeostasis and neurotransmitter regulation. Although this study specifically focused on PTZ-induced epilepsy, the oxidative and inflammatory mechanisms modulated by tDCS are not unique to this model. Given that such pathophysiological processes are shared among various forms of epilepsy, the neuroprotective potential of tDCS observed here may have broader translational relevance for other epileptic conditions.

However, this study has certain limitations. The relatively short duration of the tDCS intervention and the lack of electrophysiological validation may restrict the interpretation of neuronal excitability changes. Future research should include extended stimulation protocols and electrophysiological recordings to further elucidate the mechanistic basis of tDCS-induced neuroprotection.

## 5. Conclusions

This research demonstrates that anodal transcranial direct current stimulation (tDCS) confers substantial neuroprotective benefits in experimental models representing both acute and long-term temporal lobe epilepsy. Behavioral assessments revealed that tDCS improved cognitive performance, particularly in recognition and spatial memory tasks, while biochemical analyses showed restoration of antioxidant defenses and reduction in lipid peroxidation. Furthermore, tDCS attenuated neuroinflammatory responses, as evidenced by decreased levels of TNF-α and IL-1β, and reduced astrocytic activation and nNOS expression in the hippocampus. Histopathological findings corroborated these results, indicating diminished neuronal degeneration and vascular alterations in tDCS-treated groups. Collectively, these outcomes highlight tDCS as a promising and well-tolerated adjunctive therapy for epilepsy, capable of mitigating seizure-related oxidative stress, inflammation, and cognitive impairments. Future studies are warranted to further elucidate the underlying mechanisms, particularly regarding hippocampal glutamatergic and GABAergic signaling and their contributions to tDCS-mediated neuroprotection.

## Figures and Tables

**Table 1:** Scoring of histopathological, immunohistochemical and immunofluorescence findings of acute and chronic groups.

	Control	Acute Epilepsy	Acute Epilepsy+ tDCS	Chronic Epilepsy	Chronic Epilepsy+ tDCS
**Degeneration in neurons**	−	+++	++	++	+
**Necrosis of neurons**	−	++	−	+	−
**Hyperemia in the veins**	−	+++	++	++	++
**GFAP**	−	+++	++	++	+
**nNOS**		+++	++	++	+

Scale bar: 50 µm. Immunostaining intensity was graded as none (−), mild (+), moderate (++), or severe (+++) according to semiquantitative evaluation criteria.

## Data Availability

All data supporting the findings of this study are included within the manuscript. Additional datasets are available from the corresponding author upon reasonable request.

## Abbreviations

ANOVA	Analysis of Variance
Ca^2+^	Calcium ion
DAB	3,3′-Diaminobenzidine
ELISA	Enzyme-Linked Immunosorbent Assay
FITC	Fluorescein Isothiocyanate
GABA	Gamma-Aminobutyric Acid
GFAP	Glial Fibrillary Acidic Protein
GLU	Glutamate
GSH	Glutathione
HE	Hematoxylin–Eosin
i.p.	Intraperitoneal
IL-1β	Interleukin-1 Beta
MDA	Malondialdehyde
Na^+^	Sodium ion
nNOS	Neuronal Nitric Oxide Synthase
NMDA	N-Methyl-D-Aspartate
NO	Nitric Oxide
NOR	Novel Object Recognition
OF	Open Field
PTZ	Pentylenetetrazole
SEM (or s.e.m.)	Standard Error of the Mean
SOD	Superoxide Dismutase
SPSS	Statistical Package for the Social Sciences
TAS	Total Antioxidant Status
TLE	Temporal Lobe Epilepsy
TNF-α	Tumor Necrosis Factor-Alpha
tDCS	Transcranial Direct Current Stimulation
TOS	Total Oxidant Status
Y-Maze	Y-Shaped Maze Test
